# Technical notes on the placement of cerebral microdialysis: A single center experience

**DOI:** 10.3389/fneur.2022.1041952

**Published:** 2023-01-09

**Authors:** Joseph A. Falcone, Jefferson W. Chen

**Affiliations:** Department of Neurosurgery, University of California, Irvine, Orange, CA, United States

**Keywords:** cerebral microdialysis, traumatic brain injury, multimodal brain monitoring, brain metabolism, neurocritical care

## Abstract

**Background:**

Cerebral microdialysis enables monitoring of brain metabolism and can be an important part of multimodal monitoring strategies in a variety of brain injuries. Microdialysis catheters can be placed in brain parenchyma through a burr hole, a cranial bolt, or directly at the time of an open craniotomy or craniectomy. The location of catheters in relation to brain pathology is important to the interpretation of data and guidance of interventions.

**Methods:**

Here we retrospectively review the use of cerebral microdialysis at a US Regional Medical Center between March 2018 and February 2022 and provide detailed descriptions and technical nuances of the different methods to place microdialysis catheters.

**Results:**

Eighty two unique microdialysis catheters were utilized in 52 patients. 35 (42.68%) were placed *via* a quad-lumen bolt and 47 (57.32%) were placed through craniotomies. 27 catheters (32.93%) were placed in a perilesional location, 50 (60.98%) were located in healthy tissue, and 6 (7.32%) were mispositioned. No significant difference was seen between placement by bolt or craniotomy in regard to perilesional location, mispositioning, or complications.

**Conclusion:**

With careful planning and thoughtful execution, cerebral microdialysis catheters can be successfully placed though a variety of strategies to optimize and individualize brain monitoring in different clinical settings. This paper provides a detailed guide for the various methods of catheter placement to help providers begin or expand their use of cerebral microdialysis.

## 1. Introduction

The principles of cerebral microdialysis as a research technique have been extensively reviewed (1, 2). In short, this technology uses the principle of diffusion to drive small molecules that have high concentration in brain tissue across a semipermeable membrane at the tip of a flexible dual lumen catheter with continuous flow of isotonic dialysate solution which can be serially collected and analyzed (3). In common practice, this technology is used to measure small molecules in the extracellular space to give insight into brain metabolism, oxidative stress, and excitotoxicity at a cellular level (4).

Since its introduction, cerebral microdialysis has been used extensively in the clinical management of multiple brain pathologies, particularly traumatic brain injury (TBI) and aneurysmal subarachnoid hemorrhage (SAH). In TBI, microdialysis has been used for prognostication (5–7), to detect the development of secondary injury (8, 9), and to optimize medical management (10–13). Likewise, in SAH it has been used for prognostication of high grade SAH patients (14), to detect the development of delayed cerebral ischemia (15–17), and optimize management (18–20).

Since the data from one catheter represent the cellular metabolism of a small area of brain tissue, knowing the location of the catheter is vital to interpreting the data. Tissue considered “at-risk,” such as that adjacent to a hematoma or edema in TBI, or in a particular vascular territory at risk for vasospasm in SAH, can therefore be targeted.

Despite now extensive experience, the clinical utility of cerebral microdialysis remains a subject of debate (21, 22). A large part of the uncertainty likely relates to the heterogeneity of the pathologies being monitored, the variability in placement strategies, and the challenge of interpreting data which can be widely variable depending on the clinical scenario and the tissue being monitored. The greatest utility for modern cerebral microdialysis is often thought to be a component of a multimodal monitoring strategy with individualized patient management (4, 23, 24).

Here we describe our experience using cerebral microdialysis and technical notes on methods of optimizing catheter placement. We discuss placement both through a craniotomy and though a bolt, and test our null hypothesis that when chosen thoughtfully, neither strategy has significantly higher rate of catheter misplacement or malfunction.

## 2. Materials and methods

Retrospective chart review was performed of a prospectively collected cohort of patients who underwent monitoring with cerebral microdialysis at a single US Regional Medical Center between March 2018 and February 2022. This is an American College of Surgeon Level 1 trauma center and a JCAHO (Joint Commission on Accreditation of Healthcare Organizations) certified Comprehensive Stroke Center. All research was conducted with approval and in accordance with our center's Institutional Review Board. Clinical information including the nature of the brain injury as well as the mechanism of injury were determined by retrospective review of medical records and imaging.

**Figure 1:**
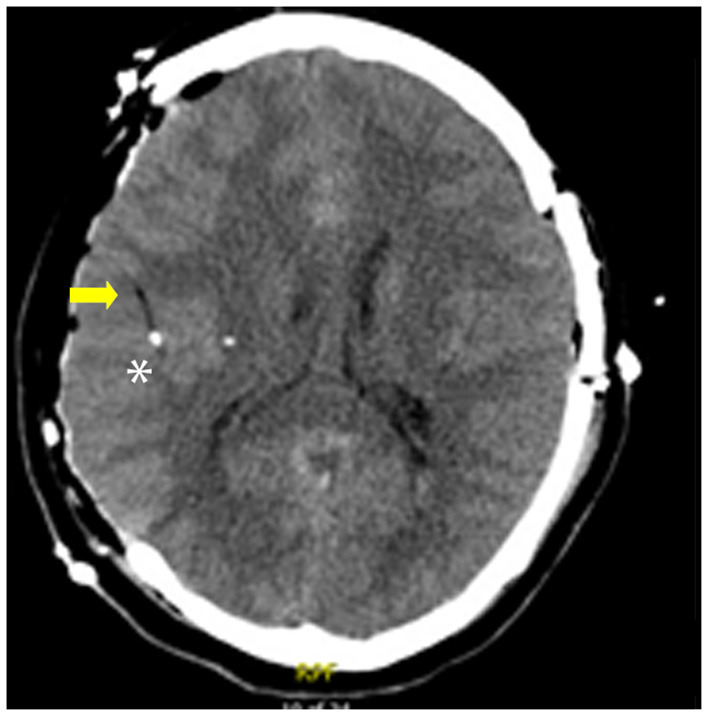
Example of the gold probe tip on CT a patient who underwent hemicraniectomy. Note the hypodense linear catheter (arrow), and hyperdense gold filament tip (*).

Classification of the location of the catheter tip was retrospectively determined by review of post-placement CT. The 70 Microdialysis Brain and Bolt Catheters include a gold thread at the tip which allows easy visualization on CT (25). This appears as a small hyperdensity, and the catheter appears as a linear hypodense structure (Figure 1). It is our practice to routinely obtain CT imaging after catheter placement to confirm final catheter location and guide interpretation of data. While the catheters are compatible with Magnetic Resonance Imaging, we did not routinely use this modality for localization given the easier availability of CT at our institution. Perilesional location of catheter was defined as a catheter tip within 1 cm of abnormal tissue on CT, determined either as hypodense tissue indicating edema or infarct, or hyperdensity indicating hemorrhage or contusion. Healthy tissue was that which on CT had radiographically normal appearance. Mispositioned probes were those that did not provide usable data due to position either too superficial, within the ventricle or periventricular tissue or within a hematoma. The decision for monitor placement, position of the catheters, and duration of monitoring were at the discretion of the managing attending neurosurgeon and neurocritical care team. All surgical and critical care management was performed as standard of care.

Statistical analysis of data was performed using IBM SPSS Statistics (Version 28.0, IBM Corp, Armonk, NY) with Chi-Squared test of independence used to compare data for non-continuous and categorical variables and a *p*-value < 0.05 considered statistically significant.

A variety of placement strategies were used for the implantation and securing of the microdialysis catheters, including direct implantation in the brain through either a craniotomy or craniectomy in an operating room or placement through a twist drill hole or skull-mounted bolt system either at the time of surgery or independently at bedside. At our facility, this choice is determined by the patients' need for operative intervention. If no craniotomy is planned, then bedside placement is the default strategy. Patients requiring operative intervention preferentially have catheters placed through the craniotomy, with targets determined by the underlying pathology. Intended targets are typically chosen 2–3 cm into the parenchyma to avoid catheter dislodgement if too superficial, or intraventricular location if too deep. Patients who undergo bedside placement but later require surgery due to evolution of their condition may have additional catheters placed through the craniotomy in addition to those originally placed bedside.

Infection risk with intracranial monitors has been shown to be low (26, 27), and routine prophylaxis is not generally recommended (28, 29). As such we do not utilize prophylactic antibiotics specifically for microdialysis catheters.

## 3. Results

A total of 82 unique microdialysis catheters were utilized in a total of 52 patients. Patients were 63.46% male with average age 53.12 years. The primary pathologies leading to need for monitoring and the mechanisms of injury are detailed in Table 1. Microdialysis was typically used as a component of a multimodal monitoring strategy in combination with intracranial pressure monitoring in 47 (90.38%), brain temperature in 43 (82.69%), brain oxygenation in 41 (78.85%) and cerebral perfusion in 40 (76.92%) patients. Average duration of monitoring was 4.06 days (SD 2.13 days).

**Table 1:** Pathologies and mechanisms of injury of the 52 patients undergoing monitoring with cerebral microdialysis.

**Primary pathology**	***n***	**%**
Acute SDH	14	26.92
Chronic SDH	1	3.85
Traumatic ICH	15	26.92
Ischemic stroke	4	7.69
Spontaneous ICH	13	25
DAI	2	3.85
Aneurysmal SAH	3	5.77
**Mechanism of injury**	***n***	**%**
Ground level fall	9	17.31
Fall from height	4	7.69
Motor vehicle accident	14	26.92
Aneurysm	3	5.77
Ischemic stroke	4	7.69
Hypertensive hemorrhage	13	25
Other head trauma	4	7.69
Coagulopathy	1	1.92

SDH, subdural hematoma; ICH, intracerebral hematoma; DAI, diffuse axonal injury; SAH, subarachnoid hemorrhage.

Fifty one patients (98.07%) underwent neurosurgical intervention, of which 19 (36.54%) underwent decompressive hemicraniectomy, 13 (25.00%) underwent craniotomy, and 19 (36.54%) underwent minimally invasive hematoma evacuation.

Of the individual microdialysis catheters, 35 (42.68%) were secured with a bolt, all of which were Hemedex Quad Lumen Bolts (Hemedex, MA, USA), and 47 (57.32%) were placed through craniotomies. 13 patients (25.00%) had catheters placed both through bolts and craniotomies. Of these 6 (11.54%) bolts were ipsilateral and 7 (13.46%) were contralateral to the craniotomy. One patient underwent a period of monitoring before a new hemorrhage required additional surgery including removal of the original catheters and placement of two new microdialysis catheters on the side of the craniotomy.

### 3.1. Placement through craniotomy

**Figure 2:**
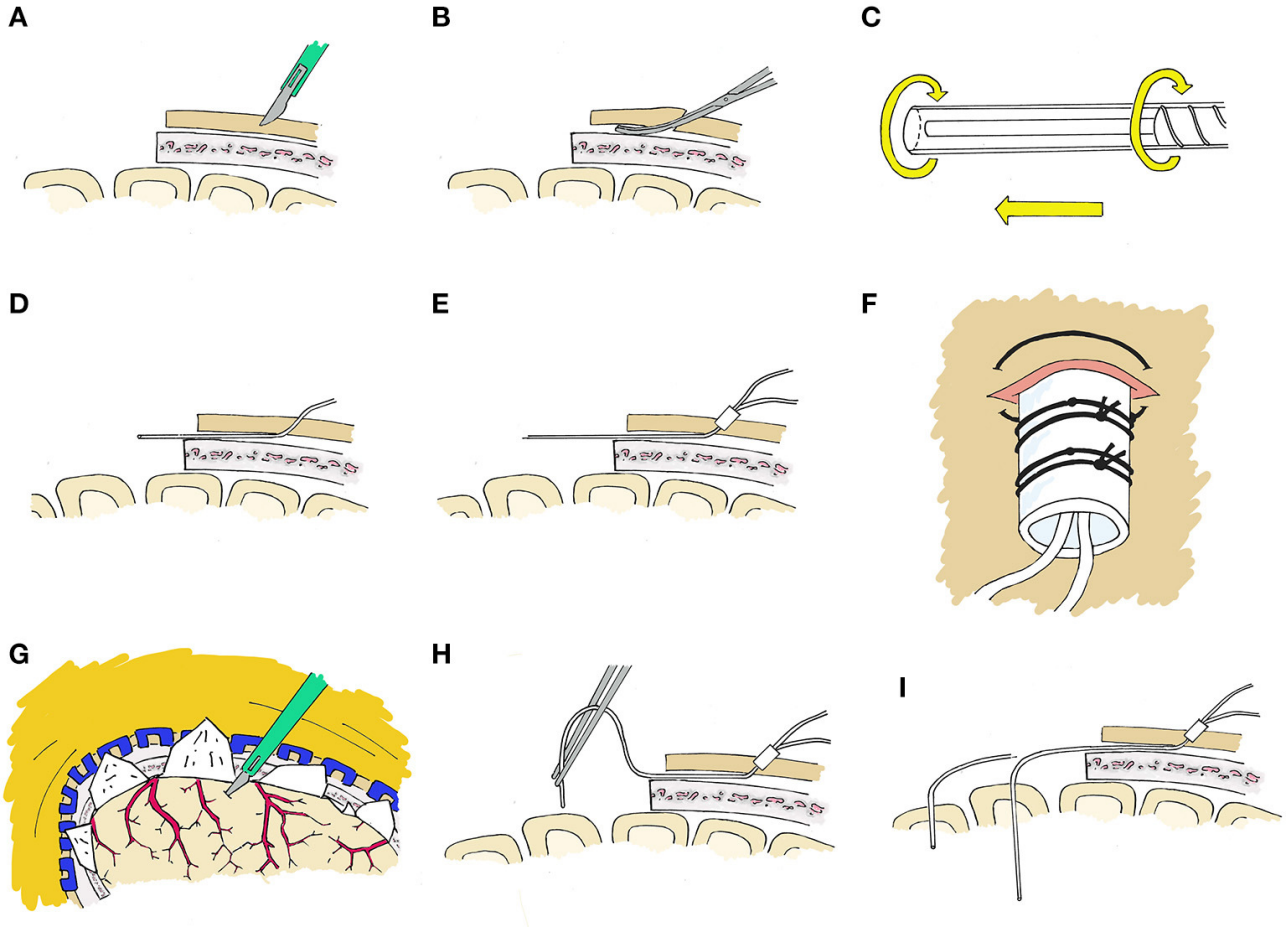
Stepwise placement through craniotomy. **(A)** First, a 2 mm incision is made with a 15 blade at the intended exit site. **(B)** An instrument such as a Kelly clamp is used to tunnel beneath the galea and create a pathway for the microdialysis catheter. **(C)** The protection tube will be removed from the catheter after tunneling, however we have found it best to loosen the protection tube by partially unscrewing the base prior to tunneling to facilitate the process. **(D)** The catheter is then tunneled through the subgaleal tract toward the craniotomy site, after which the protection tube is removed. **(E)** The fixation cuff is positioned firmly within the skin opening to serve as a plug to occlude the exit site. **(F)** The fixation cuff is then secured with 4-0 nurolon suture (Ethicon, NJ, USA). **(G)** The site of corticectomy is chosen to avoid surface vessels and after cauterization of the pia with bipolar cautery, a 1mm corticectomy is made with an 11 blade. **(H)** The catheter is then inserted perpendicular to the cortical surface. **(I)** The catheter length from tip to fixation cuff is a set length, and the depth which the probe tip sits is dependent on the length tunneled beneath the galea and the distance of the corticectomy from the edge of the craniotomy and this must be taken into consideration.

**Figure 3:**
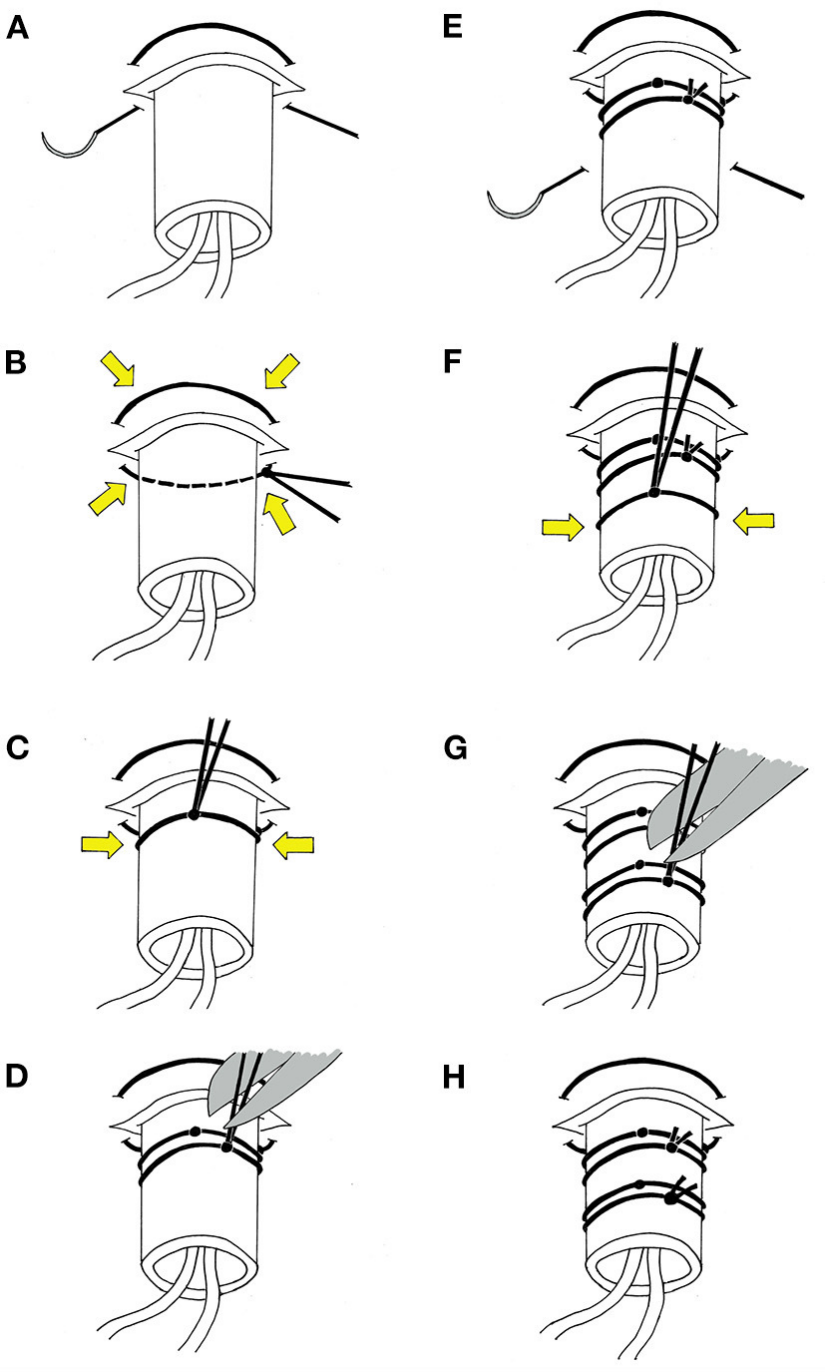
Stepwise schematic of suturing method. **(A)** With the fixation cuff lodged within the skin opening, a 4-0 Nurolon suture (Ethicon, NJ, USA) is threaded in a purse-string fashion around the base of the cuff. **(B)** The positioning of the suture sites is chosen such that when secured, the suture tightens around the skin opening and creates a watertight seal around the cuff. **(C)** The ends of the suture are then wrapped around the fixation cuff and tied. Care should be taken at this point to ensure the suture is tight enough to prevent egress of CSF through the cuff, however not so tight as to preclude flow of dialysate though the catheters. **(D)** The ends of the suture are wrapped around the cuff once again, tied, and ends trimmed. **(E)** A second 4-0 Nurolon suture is then threaded under the cuff slightly distal to the skin exit site to provide a second point of fixation. **(F)** This is secured, again with consideration to tightness. **(G)** The ends of the suture are wrapped around the cuff once again, tied, and ends trimmed. **(H)** The end result is a watertight seal around the cuff to prevent leakage of CSF while also providing two points of fixation to guard against dislodgement.

At the time of a craniotomy or craniectomy, a 70 Microdialysis Brain Catheter (CMA microdialysis AB) can be directly implanted in the brain parenchyma. This is done by a tunneling process, depicted in Figure 2. An area around the craniotomy must be prepared in a sterile manner wide enough to accommodate the exit site, and planning for this from the beginning of surgery is essential. Once tunneled, the catheter is secured by suturing the fixation cuff to the skin. We have developed a method of securing the cuff (Figure 3) using two 4-0 Nurolon sutures (Ethicon, NJ, USA) which provides watertight seal to prevent CSF egress and resultant infection risk, as well as two points of fixation to guard against catheter dislodgement, which can occur during routine nursing care or with patient movement. The catheter tip is fragile and can be broken by excess manipulation, so care should be taken with this regardless of placement strategy.

Placement through a craniotomy allows clear visualization of the cortex and vasculature, and the site of the corticectomy can be selected to avoid sulci and large blood vessels. the location of the corticectomy in relation to the skin exit site must be taken into consideration, as shorter distances between these will result in deeper localization of the catheter tip, give the fixed catheter length (Figure 2I).

After catheter placement the bone flap may remain off or be replaced as indicated by the underlying pathology. If the bone flap is replaced, care must be taken to ensure there is sufficient space at the bone edge to allow easy removal of the catheter.

### 3.2. Placement at bedside

Patients not requiring a craniotomy may have indication to place microdialysis at the bedside in the Intensive Care Unit. This can be done either through a cranial bolt, or through a twist-drill burr hole and secured to the skin.

There are multiple bolt devices designed to secure brain monitors to the skull with bedside placement. These include double or triple lumen bolts (Integra Neuroscience, NJ, USA) and a Quad Lumen Bolt (Hemedex, MA, USA), with multiple ports permitting either additional brain monitoring probes, such as for intracranial pressure or brain tissue oxygenation, or multiple microdialysis probes.

**Figure 4:**
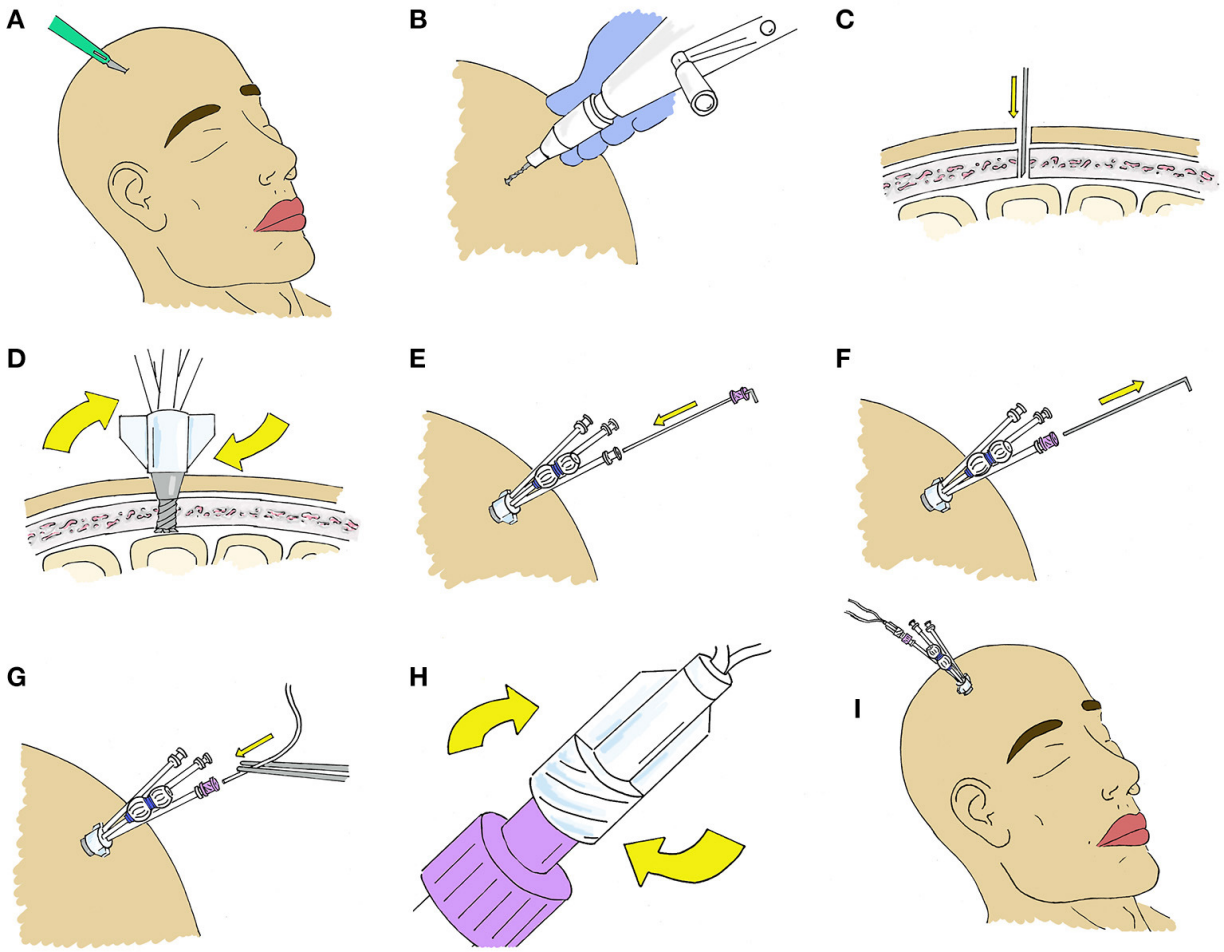
Stepwise placement at bedside through a Quad Lumen Bolt (Hemedex, Cambridge, US). **(A)** A small incision is made at the location of desired probe placement to minimize skin trauma. **(B)** A standard twist drill is used to drill a burr hole. The size of the drill bit depends on which bolt is being used and is typically included in the Bolt kit. **(C)** A durotomy is made using an 11 blade or 18-gauge needle. **(D)** The bolt is screwed into the burr hole. It should be deep enough to be firmly attached to the bone. **(E)** The sensor introducer is advanced through the desired bolt lumen where it is secured to the luer fitting and **(F)** the stylet is removed. **(G)** The microdialysis catheter is then advanced and **(H)** secured to the introducer. **(I)** After placement of microdialysis catheters, other monitors may be placed though the additional lumen if desired.

A microdialysis catheter designed specifically to work in coordination with such devices is available (70 Microdialysis Bolt Catheter, CMA microdialysis AB), and contains a Luer lock in place of a fixation cuff. When secured, this places the tip of the catheter 2.5 cm deep to the inner table of the skull, and typically in the white matter. Specifics for placement of the intracranial access bolt will differ depending on the device used, however all follow a similar pattern (Figure 4).

**Figure 5:**
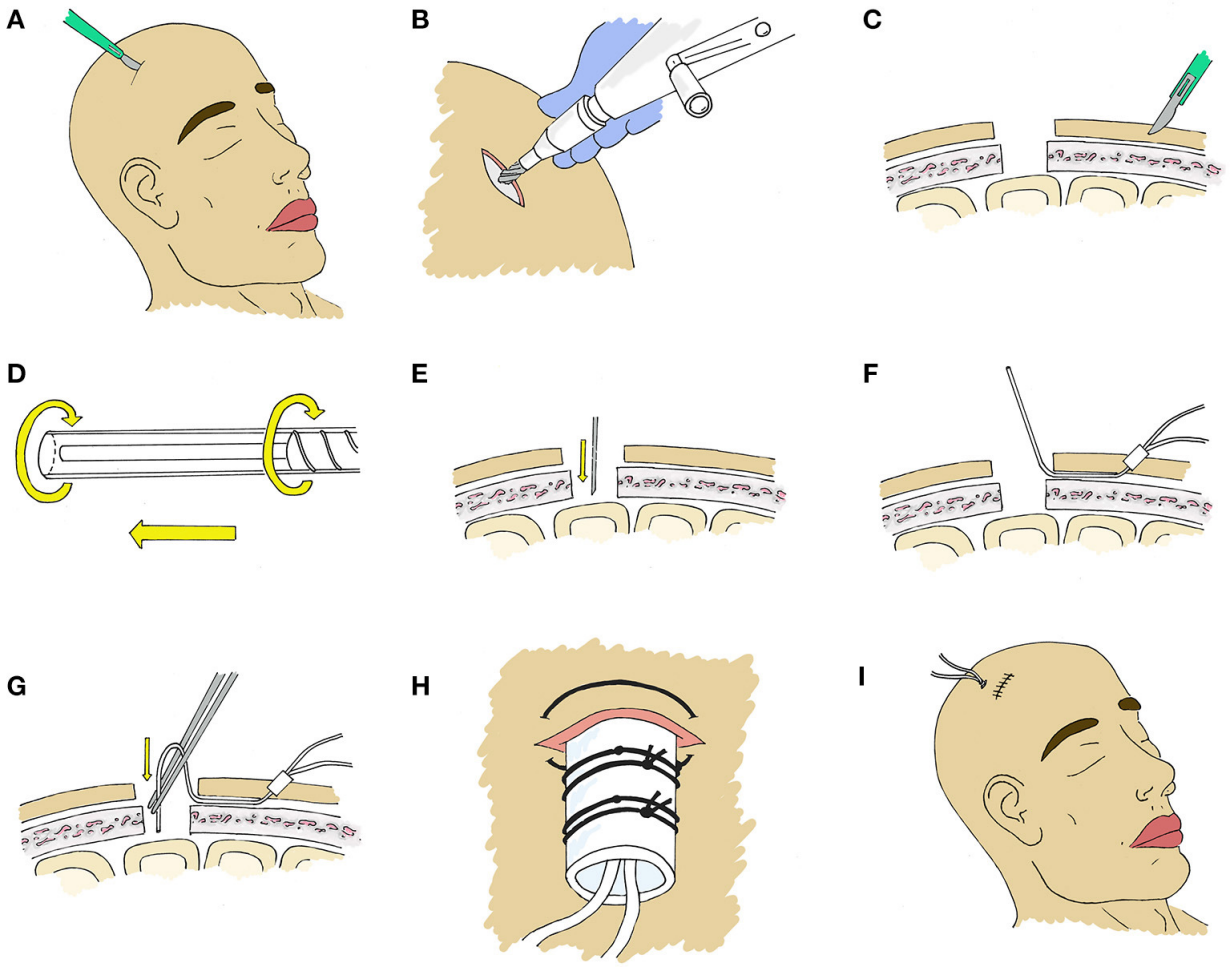
Stepwise placement at bedside through a burr hole. **(A)** An incision ~3 cm is made at the location of desired probe placement. **(B)** A burr hole is created using a standard twist drill. A larger drill bit can be used if placement of multiple probes is desired, however a smaller bit will provide adequate access for a single microdialysis catheter. **(C)** A 2 mm incision is made with a 15 blade at the intended exit site and an instrument such as a Kelly clamp is used to tunnel beneath the galea toward the incision to create a pathway for the microdialysis catheter. **(D)** The protection tube will be removed from the catheter after tunneling, however we have found it prudent to loosen the protection tube by partially unscrewing the base prior to tunneling to facilitate the process. **(E)** An 18 guage needle or 11 blade is used to create a small corticectomy within the burr hole. **(F)** The catheter is then tunneled through the subgaleal tract toward the craniotomy site, positioning the fixation cuff as a plug in the skin opening, after which the protection tube is removed. **(G)** The catheter is then inserted perpendicular to the cortical surface. **(H)** The fixation cuff is then secured with 4-0 nurolon suture (Ethicon, NJ, USA). **(I)** The incision is then closed in the usual fashion with sutures or staples.

Alternately, bedside placement is possible by percutaneous placement with a twist-drill hole (Figure 5) with a 70 Microdialysis Brain Catheter (CMA microdialysis AB). The twist-drill hole is created at bedside in the standard fashion with a twist drill, and the catheter is then tunneled to this in a similar fashion to when placed by a craniotomy. The fixation cuff is similarly secured with two 4-0 Nurolon sutures. This avoids the requirement of a specialized bolt system and can be performed with equipment commonly available in standard Cranial Access sets.

This strategy can also be used to place multiple intracranial monitors, either through the same large twist-drill hole or adjacent smaller holes, or to place a microdialysis catheter at the time of placing an External Ventricular Drain. If multiple monitors are used in this fashion, care should be taken to angle the microdialysis catheter away from other probes to avoid placement in tissue with insertional trauma from larger probes, as well as to avoid coagulating the surface at the site of entry, which may affect the resultant data.

### 3.3. Assessment of individual catheters

Twenty seven catheters (32.93%) were placed in a perilesional location, 50 (60.98%) were located in healthy tissue, and 6 (7.32%) were poorly positioned such that no useful data could be obtained.

While there was a trend toward more mispositioned catheters in the group placed by bolt, we did not see significant difference between the groups in catheter location or mispositioning (Table 2). Complications observed include one patient (1.22%) with microdialysis placed by craniotomy who had a catheter pulled out during nursing care. Two patients (3.8%), both with monitors including microdialysis placed by Quad Lumen bolt developed new hemorrhage related to the monitors, one of which required operative intervention. One patient (1.22%) had malfunction of the microdialysis device requiring replacement. We saw no cases of intracranial infection attributable to microdialysis.

**Table 2:** Comparison of catheter location and complications between placement with a bolt or a craniotomy.

	**Bolt**	**Craniotomy**	***p***
Perilesional	9	17	0.314
Healthy tissue	22	28	0.763
Mispositioned	4	2	0.217
Dislodgement	0	1	0.385
Hemorrhage	2	0	0.097
Device malfunction	0	1	0.385

## 4. Discussion

The consensus statement from the 2014 International Microdialysis Forum (4) clarified expert opinion recommendations for the placement of microdialysis catheters. In patients with diffuse traumatic injury without a focal lesion, placement in the right frontal region is recommended to provide a global picture of brain metabolism in a relatively safe brain region. The presence of a focal lesion introduces multiple options for placement, and the goal of monitoring must be taken into account.

Early microdialysis investigations showed that in TBI with focal contusions, the perilesional tissue has impaired metabolism compared to normal tissue (10, 25, 30). This perilesional tissue is also more sensitive to metabolic perturbations or changes in perfusion pressure (10, 31), and can show deterioration prior to increases in intracranial pressure (32) or the development of focal secondary injuries (33). This therefore represents a penumbra which is potentially at risk.

Assessment of global metabolism is recommended as a primary goal by the 2014 consensus statement, and placement in normal ipsilateral tissue is still recommended, though contralateral placement is also an option, e.g., if no clearly normal tissue is accessible ipsilaterally. Perilesional placement is considered an option if the goal is to monitor and optimize at-risk tissue. The possibility of multiple monitors is also addressed with the option of one catheter in normal tissue and one catheter in a perilesional location. Given that a majority of the variability in cerebral microdialysis data is related to individual patient factors (22), the interpretation of data is difficult when comparing to universal standard values, and values from normal tissue can be seen as an important internal control.

At our institution, we have found the combination of normal tissue and perilesional location most useful to interpret data within the context of a multimodal monitoring strategy, and try to employ this when focal lesions are present. Care must be taken with the perilesional catheter to place it in tissue that remains viable, as opposed to tissue too contused or infarcted to salvage.

In this manuscript we have described several strategies by which microdialysis can be placed, including through a craniotomy, through a bolt, and through a bedside twist-drill hole. No significant difference was seen between these methods for complications or device malfunctions.

Placement of microdialysis during an otherwise indicated craniotomy has several advantages, and we have found minimal increase in operative time once a surgeon is familiar with the technique. The primary advantage is the clear visualization of the cerebral cortex. This ensures that the corticectomy can be made in a location where large cortical vessels can be protected. Additionally, if any cortical bleeding does occur, the open visualization allows for easy hemostasis, either with bipolar cautery or hemostatic material. The wider field and greater access to the brain surface also provides greater freedom in directing the angle and depth at which catheters are placed, with the ability to better target specific pathology. Additionally, patients with traumatic brain injury often have contusions which may be visible from the surface, and visualization of this injured tissue can help to optimize placement. With the method of securing the catheters we have described we have not seen a significantly increased rate of catheter dislodgement compared to bolt placement (Table 2).

Placement at bedside is an option for patients not requiring a craniotomy, or when monitoring of tissue contralateral to a craniotomy is desired. Placement through a twist-drill burr hole does not require specialized equipment beyond the microdialysis catheter itself and can be performed with supplies commonly found in commercially available cranial access kits. Multiple monitors can be placed though this strategy, however angling the catheters away from each other to avoid artifact from insertional trauma is difficult. The multi-lumen bolts which are commercially available are designed to angle the monitors in order to avoid this and have become the standard for bedside placement of multimodal monitors at our institution.

While visualization of the cortex is not possible with these methods, the placement is typically limited to regions known to have minimal cortical vessels which are considered safe for blind access, such as Kocher's point, and the rate of hemorrhage with bolt-mounted intracranial monitors has been shown to be low for both single-lumen (34) and multiple-lumen monitors (35).

The primary disadvantage of this method is the limited ability to target the final position of catheters, however placement at Kocher's Point is ideal for monitoring overall brain metabolism in diffuse TBI or DAI. This position is also ideal for targeted monitoring of several specific pathologies. Frontal contusions and basal ganglia hemorrhages typically have a region of perihematomal edema which standard placement at Kocher's point targets well. Additionally, in SAH this location is typically a watershed zone between the MCA and ACA territories, and as such may be useful for vasospasm monitoring.

### 4.1. Insertional effects

The effect of insertional trauma on initial microdialysis data should also be considered. The 2004 (36) and updated 2014 (4) microdialysis consensus meetings recommended waiting 1–2 h from placement to consider data reliable. However, in animal models, while glucose and pyruvate achieved a steady state immediately, lactate and glycerol were seen to peak immediately after implantation and reach a steady state after ~4 h (37), indicating that the trauma of placement causes initial tissue damage which can affect initial data over a longer period of time than previously appreciated.

### 4.2. Limitations

This study has several limitations that must be considered. The retrospective format limits conclusions about complication rates, as it is difficult to retrospectively attribute causality of complications such as infection or hemorrhage, and some complications may be missed if they were not clearly noted in the medical record. This also limits assessment of how cerebral microdialysis data were used, as the roles these data played in clinical decision making, especially within the context of a multimodal monitoring program, is not always apparent in chart review. Prospective studies will therefore be essential to progress the cerebral microdialysis field.

This study was not designed or powered to assess impact of microdialysis on any clinical outcomes. The clinical interpretation of the microdialysis results is highly dependent on the probe location, the clinical scenario, and other multimodal monitors, which remains an area of active research and debate and is beyond the scope of this paper. Additionally, the presence in gray or white matter may impact the metabolic picture, though this also remains an area or research (38).

The expenses of starting a program and laboratory requirements can hinder the institution of microdialysis monitoring at smaller and more economically challenged centers, however even community based hospitals have demonstrated the ability to run successful microdialysis programs (24).

The placement of catheters in this study was done under supervision of an attending neurosurgeon experienced in their use. A learning curve may lead to problems early on for a surgeon new to microdialysis, such as device malfunction or suboptimal catheter placement. It is our hope that the insights provided here may help minimize this.

## 5. Conclusions

Microdialysis offers a unique means of monitoring focal brain tissue metabolism, which may have a role in the management of multiple forms of brain injury, particularly as part of a multimodal monitoring strategy. Interpretation of microdialysis data is highly dependent on the location of the catheter probe tip in the brain parenchyma as well as the underlying pathology. Different strategies can be used to place microdialysis catheters including through a craniotomy, a bolt, and a twist drill hole. Each of these has unique clinical advantages and shortfalls, as well as procedural nuances. This paper provides a detailed guide for the various methods of catheter placement to help providers begin or expand their use of cerebral microdialysis.

## Data availability statement

The original contributions presented in the study are included in the article/supplementary material, further inquiries can be directed to the corresponding author.

## Ethics statement

Ethical review and approval was not required for the study on human participants in accordance with the local legislation and institutional requirements. Written informed consent for participation was not required for this study in accordance with the national legislation and the institutional requirements.

## Author contributions

JF reviewed and collected patient data, drafted manuscript, and generated images. JC supervised patient care and collected patient information, assisted in editing of manuscript, and editorial contributions to images. Both authors contributed to the article and approved the submitted version.
